# Erdheim‐Chester Disease with atrial mass and perinephric calcification

**DOI:** 10.1002/ccr3.1258

**Published:** 2017-11-02

**Authors:** Michel Villatoro‐Villar, Matthew J. Koster

**Affiliations:** ^1^ Division of Cardiovascular Diseases and the Gonda Vascular Center Mayo Clinic Rochester Minnesota; ^2^ Division of Rheumatology Mayo Clinic College of Medicine and Science Rochester Minnesota

**Keywords:** Erdheim‐Chester Disease, non‐Langerhans cell histiocytosis, perinephric dystrophic calcification, pseudotumoral infiltration of the right atrium

## Abstract

Erdheim‐Chester Disease is a multi‐systemic condition characterized by non‐Langerhans histiocytic infiltration. Cardiovascular involvement with pseudotumoral infiltration of the right atrium is present in approximately one‐third of patients and may be asymptomatic. Although retroperitoneal fibrosis is common, perinephric dystrophic calcification has not been previously described.

A 71‐year‐old male presented to the emergency department with a new‐onset pericardial effusion, successfully treated with pericardiocentesis. A right atrial mass was identified on magnetic resonance imaging of the chest (Fig. 1, panel A, T_2_ weighted, fast field echo image, horizontal long‐axis view. Arrows) and found to have increased ^18^F fluorodeoxyglucose (FDG) uptake on fusion positron emission tomography (PET) – computed tomography (CT) (Fig. 1, panel B, axial view, and panel C, coronal view, arrows). Biopsy of the atrial mass demonstrated histiocytic infiltration, positive for CD68, negative for S100 and CD1a, compatible with Erdheim‐Chester Disease (ECD). BRAF V600E mutation testing by capture‐based next‐generation sequencing was negative 1, 2. CT of the abdomen disclosed extensive calcification in the main branches of the aorta, retroperitoneal fibrosis, as well as perinephric dystrophic calcification (Fig. 1, panel D, axial view; and panel E, coronal view). Renal biopsy performed was also consistent with ECD, showing evidence of CD68 (+), CD1a (−) histiocytic inflammatory infiltrate. In addition, dense dystrophic calcification was histologically observed. Treatment was initiated with prednisone 60 mg/day and anakinra 100 mg subcutaneous every 48 h (adjusted for renal function). A follow‐up PET‐CT revealed moderate decrease in the soft tissue density and reduction in FDG uptake in the right atrial infiltrative mass but the perinephric thickening and dystrophic calcification were unchanged (Fig. 1, panel F, axial view, and panel G, coronal view, arrows).

**Figure 1:**
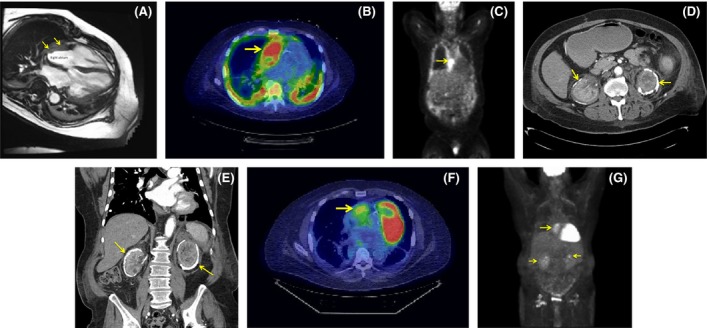
(A) Magnetic resonance imaging of the chest, T2 weighted, fast field echo image, horizontal long axis view showing an atrial mass infiltrating the right atrial wall (arrows). (B) PET‐CT, axial view and (C) coronal view, showing FDG increase uptake in the right atrial mass. (D) CT scan of the abdomen with contrast, axial view and (E) coronal view, showing severe perinephric dystrophic calcification (arrows). (F) Follow‐up PET‐CT, axial view, and (G) coronal view, reveal moderate decrease in the soft tissue density and FDG uptake in the right atrial infiltrative mass. Perinephric thickening and dystrophic calcification unchanged (arrows).

## Conflict of Interest

The Authors declare that there is no conflict of interest regarding the publication of this article.

## Authorship

MV‐V: involved in the conceptualization, preparation, writing, and review of this manuscript. MJK: involved in the conceptualization, writing, and review of this manuscript.

## References

[1] Emile, J. F. , O. Abla , S. Fraitag , A. Horne , J. Haroche , J. Donadieu , et al. 2016 Revised classification of histiocytoses and neoplasms of the macrophage‐dendritic cell lineages. Blood 127:2672–2681. 26966089 10.1182/blood-2016-01-690636 PMC5161007

[2] Estrada‐Veras, J. I. , K. J. O'Brien , L. C. Boyd , R. H. Dave , B. Durham , L. Xi , et al. 2017 The clinical spectrum of Erdheim‐Chester disease: an observational cohort study. Blood Adv. 1:357–366. 28553668 10.1182/bloodadvances.2016001784 PMC5446206

